# Supplementary material for: Soil-borne fungi alter the apoplastic purinergic signaling in plants by deregulating the homeostasis of extracellular ATP and its metabolite adenosine
Source: eLife. 2023 Nov 23;12:e92913. doi: 10.7554/eLife.92913 (PMC10746138; doi:10.7554/eLife.92913)
Supplement: Supplementary file 1. [file elife-92913-supp1.docx]

**Supplementary File 1 Primers used in this study**

**For qRT-PCR**

| **gene** | **locus** | **sequence 5‘-3‘** | **reference** |
| --- | --- | --- | --- |
| *AtGAPDH* | At1g1344 | F – TCTCGATCTCAATTTCGCAAAA  R – CGAAACCGTTGATTCCGATTC | Czechowski et al., 2005 |
| *AtDORN1* | At5g60300 | F – TGGAGTTTGTCAGGTCCATCG  R – CTGAGGATCTTCTGCAGGCAA | Choi et al., 2014 a |
| *AtENT3* | At4g05120 | F – GGTCCGGAGCAGAATGCGTT  R – GGTTACAAGCAAAGAATATGAAAGC | Traub et al., 2007 |
| *AtNSH3* | At5g18860 | F – AAGCCGAAAATGAGAGCCGA  R – TTTGTTTGCCGCGCAGTTTA | in this study |
| *AtWRKY45* | At3g01970 | F – GGAGGGAAGATGTGCATTTGTG  R – GAACAATCCATTCCCCAGGAG | Kesten et al., 2019 |
| *AtWRKY53* | At4g23810 | F – GCGACAAGACACCAGAGTCA  R – ACCGTTGGATTGAACCAGTC | Masachis et al., 2016 |
| *At1g51890* | At1g51890 | F – CTAGCCGACTTTGGGCTATC  R – CCAGTTTGTTCTGTAATACTCAGG | Van der Does et al., 2017 |
| *AtPRX33* | At3g49110 | F – ATCGTCCTTCTGATCTTGTTGCG  R – GCAGATCGAAATCCACTAAGACG | Arnaud et al, 2017 |
| *Foβ-tubulin* | g4360 | F – AATGGAGAGAGCAACACAATG  R – ATACTGAGCATGAGCCCTTTG | designed by PCB |
| *FoENT* | g4128 | F – GGCTCAACAGTACTCGAGGG  R – ACCCAGCAGGGCAAAGATAC | in this study |
| *Fo5’-NT* | g8638 | F – CAAAGACCGAGGGTCTGTCC  R – TTGCCGTCCTTGTCAAAGGT | in this study |

**To mutagenize Fo5176**

| **gene** | **locus** | **sequence 5‘-3‘** | **name** |
| --- | --- | --- | --- |
| 5’-NT | g8638 | TGCAGCATGGCTGAGGC | E5’NT promoter FW |
| 5’-NT | g8638 | CAGAATGCACAGGTACACTTGTTTGATGTTGACCTACGCACAAAGAAAAG | E5’NT promoter REV overhang |
| 5’-NT | g8638 | AGGGGCTGTATTAGGTCTCGATGGTAGAAATGCATTTTGTATATATAGATGACTTACAGT | E5’NT terminator FW overhang |
| 5’-NT | g8638 | TCCCCCGTCCCACTCAA | E5’NT terminator REV |
| 5’-NT | g8638 | AAGTCTTTGATCGGCCGGG | E5’NT geno FW |
| 5’-NT | g8638 | TCCTCAGCAGATACCAAGCC | E5’NT geno WT REV |
| ENT | g4128 | CGGTGTCAAGCGGCGATG | ENT promoter FW |
| ENT | g4128 | CAGAATGCACAGGTACACTTGTTTGACGTCTACGTATCAATGTTGTTCGA | ENT promoter REV overhang |
| ENT | g4128 | AGGGGCTGTATTAGGTCTCGAACGGATTACACAATATTACATTAGAATTGGGG | ENT terminator FW overhang |
| ENT | g4128 | ACCTGCACGATCCCGGTC | ENT terminator REV |
| ENT | g4128 | TCGACGACTCTGCCACTATC | ENT geno FW |
| ENT | g4128 | TCCTATGAGTCGTTTACCCAGA | ENT geno WT REV |
|  |  | TCCTATGAGTCGTTTACCCAGA | geno mut REV |
|  |  | CCAAGTTCTTCAGCAATATCACGGG | Neo FW |
|  |  | TCTTGCAGTTCATTCAGGGCAC | Neo REV |
