# Supplementary material for: Soil-borne fungi alter the apoplastic purinergic signaling in plants by deregulating the homeostasis of extracellular ATP and its metabolite adenosine
Source: eLife. 2023 Nov 23;12:e92913. doi: 10.7554/eLife.92913 (PMC10746138; doi:10.7554/eLife.92913)
Supplement: Supplementary file 2. [file elife-92913-supp2.docx]

**Supplementary File 2. Statistical analysis of Fo5176 pSIX1::GFP root vascular penetrations in the indicated Arabidopsis genotypes.**

**Supplementary File 2A (linked to Figure 1D).**

| **Two-way RM ANOVA (Tukey test)** | **Adjusted p-value** |
| --- | --- |
| **4 dpt** | |
| control vs. 500 µM ATP | ns |
| control vs. 1 mM Ado | ns |
| control vs. 500 µM ATP + 1 mM Ado | ns |
| 500 µM ATP vs. 1 mM Ado | ns |
| 500 µM ATP vs. 500 µM ATP + 1 mM Ado | ** |
| 500 µM ATP + 1 mM Ado vs. 1 mM Ado | ns |
| **5 dpt** | |
| control vs. 500 µM ATP | ns |
| control vs. 1 mM Ado | ns |
| control vs. 500 µM ATP + 1 mM Ado | ns |
| 500 µM ATP vs. 1 mM Ado | ns |
| 500 µM ATP vs. 500 µM ATP + 1 mM Ado | *** |
| 500 µM ATP + 1 mM Ado vs. 1 mM Ado | ns |
| **6 dpt** | |
| control vs. 500 µM ATP | * |
| control vs. 1 mM Ado | ns |

| control vs. 500 µM ATP + 1 mM Ado | ns |
| --- | --- |
| 500 µM ATP vs. 1 mM Ado | ns |
| 500 µM ATP vs. 500 µM ATP + 1 mM Ado | * |
| 500 µM ATP + 1 mM Ado vs. 1 mM Ado | ns |
| **7 dpt** | |
| control vs. 500 µM ATP | ** |
| control vs. 1 mM Ado | ns |
| control vs. 500 µM ATP + 1 mM Ado | ns |
| 500 µM ATP vs. 1 mM Ado | ** |
| 500 µM ATP vs. 500 µM ATP + 1 mM Ado | **** |
| 500 µM ATP + 1 mM Ado vs. 1 mM Ado | ns |

**Supplementary File 2B (linked to Figure 2A).**

| **Two-way RM ANOVA (Tukey test)** | **Adjusted p-value** |
| --- | --- |
| **6 dpt** | |
| WT vs. *dorn1* | * |
| WT vs. *ent3* | ns |
| WT vs. *ent3nsh3* | ** |
| *dorn1* vs. *ent3* | ns |
| *dorn1* vs. *ent3nsh3* | ns |
| *ent3* vs. *ent3nsh3* | ns |
| **7 dpt** | |
| WT vs. *dorn1* | * |
| WT vs. *ent3* | ns |
| WT vs. *ent3nsh3* | ** |
| *dorn1* vs. *ent3* | ns |
| *dorn1* vs. *ent3nsh3* | ns |
| *ent3* vs. *ent3nsh3* | ns |

**Supplementary File 2C (linked to Figure 1 - figure supplement 2B).**

| **Two-way RM ANOVA (Tukey test)** | **Adjusted p-value** |
| --- | --- |
| **6 dpt** | |
| control vs. 500 µM ATP | * |
| control vs. 500 µM ATP + 500 µM Ado | ns |
| control vs. 500 µM ATP + 750 µM Ado | ns |
| control vs. 500 µM ATP + 1 mM Ado | ns |
| **7 dpt** | |
| control vs. 500 µM ATP | ** |
| control vs. 500 µM ATP + 500 µM Ado | * |
| control vs. 500 µM ATP + 750 µM Ado | 0.14 |
| control vs. 500 µM ATP + 1 mM Ado | ns |
